# Supplementary material for: Rapid turnover of life-cycle-related genes in the brown algae
Source: Genome Biol. 2019 Feb 14;20:35. doi: 10.1186/s13059-019-1630-6 (PMC6374913; doi:10.1186/s13059-019-1630-6)
Supplement: Supplementary file 3 — Protein alignments, corresponding Gblocks html file and CDS sequences for the lineage conserved gametophyte-biased genes. (ZIP 2109 kb) [file 13059_2019_1630_MOESM3_ESM.zip › Con_GA_alignments/Ec-06_010960.aa_ali.fasta-gb.htm]

Ec-06\_010960.aa.fasta.trans.aa\_ali.fasta


## Gblocks 0.91b Results

Processed file: **Ec-06\_010960.aa.fasta.trans.aa\_ali.fasta**  
Number of sequences: **2**  
Alignment assumed to be: **Protein**  
New number of positions: **482** (selected positions are underlined in blue)

```
                         10        20        30        40        50        60
                 =========+=========+=========+=========+=========+=========+
Ec-06_010960     MATAFGAPAPLPGQPGFRARPSESMARETAEMEERLSRLKTDLAVEAEMREAAGPRAGGA
MSTRG.58701.1    MTAEFGAPAPLPGQRGFGARPSESMARETAEMEERLARLKTDLAAEAEMREAAGPRKGGA
                 ############################################################


                         70        80        90       100       110       120
                 =========+=========+=========+=========+=========+=========+
Ec-06_010960     RWRSARIDRGSVRAYAKDVKIRHKQQLQKRPPHGIFAN----------------------
MSTRG.58701.1    RWRSARTDRGSVRAYAKDVKIRHKQQLHHRPPHGIFANGGKVQPQAGAGARAAAALRDEQ
                 ######################################                      


                        130       140       150       160       170       180
                 =========+=========+=========+=========+=========+=========+
Ec-06_010960     ---------------K---------------SAEQRVR--PSPAFASKEVTRWGVGDTLG
MSTRG.58701.1    QQRQQQEHLHQPAKDEMPERGIRAASHLLPQASTDQCAPAVRPSLESKEVSQWGVADTLE
                                                           ##################


                        190       200       210       220       230       240
                 =========+=========+=========+=========+=========+=========+
Ec-06_010960     WLDSLGLEKYRSVFQQNEISGTILLEVGLDDLDYMDVRVLAHRKRILKGIEDLRRGGRKK
MSTRG.58701.1    WLDSLGLGKHKDVFQQNEISGPILLEVGLDDLDYMSVRVLAHRKRLLKGIEDLRRGG---
                 #########################################################   


                        250       260       270       280       290       300
                 =========+=========+=========+=========+=========+=========+
Ec-06_010960     RALDANMPPPPAPALELLPNQTTSSTRVNRDEKGPRGEGIGEGGAAAETTSQIAPEDCGG
MSTRG.58701.1    KSINPDKPPPPAPTPILVRRRSSSSESIDRERRTEQAT-----TAVITASGQRCHENVRG
                        #######################              ################


                        310       320       330       340       350       360
                 =========+=========+=========+=========+=========+=========+
Ec-06_010960     VGRRYSPLSGGNKQHWSTIKPLSENQRDGGGL-SPTADNLADGQYDESAASSSFKDAVAE
MSTRG.58701.1    DHASLSP--GRRKQHWSTIKPLADEQTEERGYSSPLPANLADGQYDEVAAHSDFADAVAE
                 #######  ######################  ###########################


                        370       380       390       400       410       420
                 =========+=========+=========+=========+=========+=========+
Ec-06_010960     WRGEGKYS---------TTTSATEGRASSLHRPAVVGRAPLAGMPSAAEEEKYEDMQLVF
MSTRG.58701.1    WRGARKTSSTIETADTTAKTSVTQAAASSLHRPTVICRAPLAGMLDGTQEGGESPGLLGK
                 ########           #########################################


                        430       440       450       460       470       480
                 =========+=========+=========+=========+=========+=========+
Ec-06_010960     NAKPARPGLLGGREGGRAGMGEETECWVNPFSSPRIGVKGDNSQLVDLGHEPGVSSHGTE
MSTRG.58701.1    NTTRIGKGLLGGQGGDN--KGRAGECWTNPFASPRPGKLVPASEGAPLLGDRAEPDD---
                 ###############     ############################            


                        490       500       510       520       530       540
                 =========+=========+=========+=========+=========+=========+
Ec-06_010960     TLTVAGSKQQQGVREKGHDPALALDEEAEHEAFRKAVAEWNRGKNTGVTADGSGVRDGSA
MSTRG.58701.1    ------RKMRSRARRCNDFPAL---EEAEHEAFQRAVADWNRS-GRATAPDIPHSCEGGV
                        ###############   #################                  


                        550       560       570       580       590       600
                 =========+=========+=========+=========+=========+=========+
Ec-06_010960     SGRGIGGISSAQTAT-EVEIPSRRTAEAMAEELRKQMDAEHCSQAKELEEEKRALLEGLE
MSTRG.58701.1    LDQ-IDSRSCAETATVGVALPSRRTVEAMADELREQMDAEHRLQAKDLEEQKKALLMGFE
                     ###########  ###########################################


                        610       620       630       640       650       660
                 =========+=========+=========+=========+=========+=========+
Ec-06_010960     RSR------EGETKGDQLELGAPDGGTEVEEQDDDASWREREDARSVGPSSVCSSTGRSS
MSTRG.58701.1    PSRHDRHGIDGECRGNTIEQNTVCKEEQEFGESEGASCHDDLVDPNTGSSSTSSSASLPA
                 ###                                                         


                        670       680       690       700       710       720
                 =========+=========+=========+=========+=========+=========+
Ec-06_010960     GSDRQDAEAENIGCGGHDS---------SGVEIEVVESVLGADLSIEGGVSYVVDECDSG
MSTRG.58701.1    PTSSRESKDAEVWSGGWDHASSGPAFVGAGAKIEVIQSVLGANLSIERGTKYLVDEYGSG
                                              ###############################


                 
                 =====
Ec-06_010960     DEPHF
MSTRG.58701.1    DELKL
                 ##
```

```
Parameters used
Minimum Number Of Sequences For A Conserved Position: 2
Minimum Number Of Sequences For A Flanking Position: 2
Maximum Number Of Contiguous Nonconserved Positions: 8
Minimum Length Of A Block: 10
Allowed Gap Positions: None
Use Similarity Matrices: Yes
```

```
Flank positions of the 13 selected block(s)
Flanks: [1  98]  [163  237]  [248  270]  [285  307]  [310  331]  [334  368]  [380  435]  [441  468]  [488  502]  [506  522]  [545  555]  [558  603]  [690  722]  

New number of positions in Ec-06_010960.aa.fasta.trans.aa_ali.fasta-gb:  482  (66% of the original 725 positions)
```
